# Tumor biomarkers HE4 and CA125 alongside enhanced computed tomography imaging in assessing lymph node metastasis in endometrial cancer

**DOI:** 10.6026/9732063002001453

**Published:** 2024-11-30

**Authors:** Vandana Maurya, Shruti Singh, Shubham Singh, Shashi Prabha Singh, Manish Kumar Verma

**Affiliations:** 1 Department of Obstetrics & Gynaecology, Maa Vindhyavasini Autonomous State Medical College, Mirzapur, India; 2 Department of Biochemistry, Maa Vindhyavasini Autonomous State Medical College, Mirzapur, India; 3 Department of Biochemistry, Rajashri Dashrath Autonomous State Medical College Ayodhya, Uttar Pradesh, India

**Keywords:** Serum HE4, serum CA125, enhanced CT, endometrial cancer, lymph node metastasis

## Abstract

The impact of HE4 and CA125 on lymph node metastasis in endometrial cancer and evaluate the diagnostic effectiveness of these biomarkers when combined with enhanced CT imaging to predict lymph node metastasis. The objective is to examine how HE4 and CA125 influence lymph node metastasis and to assess their diagnostic utility when paired with enhanced CT imaging to predict lymph node involvement in endometrial cancer. The study included 326 patients who underwent surgery for endometrial cancer (experimental group), alongside 98 individuals without cancer (control group). A retrospective analysis was carried out to assess the diagnostic efficacy of HE4 and CA125, in combination with enhanced CT, for predicting lymph node metastasis. Levels of HE4 and CA125 were measured and compared between the experimental and control groups, as well as within the lymph node-positive and -negative groups. Significant variations in HE4 and CA125 levels were found between the endometrial cancer and control groups, and between lymph node-positive and -negative subgroups within the endometrial cancer cohort (p < 0.001). The AUC for HE4 was 0.73 (p < 0.001) in premenopausal and 0.578 (p = 0.164) in postmenopausal groups. For CA125, the AUC was 0.81 (p < 0.001) in premenopausal and 0.671 (p = 0.002) in postmenopausal groups. Cut-off concentrations to predict lymph node metastasis: Premenopausal - HE4 = 52.95 pmol/l, CA125 = 69.45 U/ml; Postmenopausal - HE4 = 69.15pmol/l, CA125 = 21.45 U/ml. Combining enhanced CT imaging with HE4 and CA125 improved diagnostic accuracy compared to individual tests. In conclusion, the study offers valuable insights into the potential usefulness of HE4 and CA125, in conjunction with enhanced CT imaging, for diagnosing and predicting lymph node metastasis in patients with endometrial cancer.

## Background:

Endometrial carcinoma (EC) is a common gynaecological cancer, of which there were 380,000 new cases worldwide in 2018 [1]. The primary treatment for endometrial cancer is surgery, and accurate assessment of preoperative condition is important in guiding the scope of surgery, especially for patients in early stages, 10.5%-14.9% of whom may experience lymph node metastasis [2]. With the gradual diversification of treatment methods such as surgery, radiotherapy, chemotherapy, and targeted therapy, lymph node resection has become important for evaluating tumor stages and guiding prognostication, whereas the impact of treatment has become relatively less important because lymph node resection does not improve OS or PFS [3- 4]. In fact, the removal of lymph nodes increases the incidence of surgical complications and sequelae in patients. Therefore, increasing attention has been paid to accurate preoperative assessment and sentinel lymph node resection rather than systemic lymph node resection in the diagnosis and treatment of endometrial cancer. Pre-judgment of lymph node metastasis in low-risk patients with lesions limited to the uterus before surgery can decrease unnecessary lymph node dissection and improve patient quality of life. The preoperative evaluation of endometrial cancer relies on imaging and tumor marker detection. Common imaging tests for endometrial cancer include CT, MRI, and PET-CT. Although PET-CT has high specificity for disease evaluation, it is expensive and difficult to apply universally. In clinical practice, MRI is commonly used to evaluate muscle layer infiltration and cervical involvement, which are valuable for assessing the local condition. CT is favoured for assessing lymph node metastasis. However, morphological examination of lymph node metastasis by CT is hindered by subjectivity according to the diagnosing physician and thus, it cannot clearly distinguish between small nodal metastasis and inflammatory hyperplasia. Reich *et al.* have reported that 54% of lymph node positive patients are diagnosed negative by CT, and 29% of lymph node negative patients are diagnosed positive by CT [5]. These results clearly indicate the relatively high false positive rate of CT diagnosis of lymph node metastasis. A meta-analysis conducted by Kayal *et al.* has reported a sensitivity of 62% and a specificity of 87% for CT diagnosis of lymph node metastasis [6]. Therefore, a satisfactory image evaluation method for lymph node metastasis of endometrial cancer is needed. Cancer antigen 125or carbohydrate antigen 125 also known as mucin 16 or MUC16 is a protein that is encoded by the gene MUC16 in humans. MUC16 is a member of the glycoprotein family of mucin.

The discovery of tumor specific biomarkers has long been a hotspot in the field of cancer diagnosis, but little progress has been made in identifying biomarkers for endometrial cancer. At present, the most widely used biomarker for endometrial cancer is CA125, a high molecular weight glycoprotein located at the cell membrane of body cavity epithelium cells. CA125 ≥35 U/mL is defined as positive. Wang *et al.* have reported that the sensitivity of CA125 in the diagnosis of lymph node metastasis in endometrial cancer is 72.2% [7], but CA125 increases are observed not only in malignant tumors, but also in some benign diseases [8]. Since Moore *et al.* discovered that HE4 is overexpressed in endometrial glands in 2008; HE4 has been studied extensively in the field of endometrial cancer. The authors detected HE4 and CA125 in 1042 patients with benign gynaecological diseases and found that the increase in HE4 levels in benign diseases was less than that of CA125 [9]. The reference value for HE4 in the United States is ≤150 pmol/L, a value also used by most countries. However, Tian *et al.* after surveying HE4 reference values in multi-centers in China in 2015 have noted that the reference value for HE4 in the Chinese population is 105.1pmol/L, which is significantly lower than that in Western populations (150pmol/L). Moreover, the authors found that age and menopause status are important factors [10]. Although researchers at the 2016 ESMO-ESGO-ESTRO meeting have proposed that HE4 is associated with lymph node metastasis, muscular invasion, staging and differentiation of endometrial cancer, no consensus has been reached regarding the appropriate cut-off value for HE4 [11]. Thus, the critical cut-off value for HE4 in endometrial cancer must be further studied. There is currently no ideal biomarker for evaluating lymph node metastasis of endometrial cancer. The assessment of lymph node metastasis in endometrial cancer holds significant clinical importance, yet there lacks an ideal evaluation method encompassing both imaging and biomarkers. This study aimed to investigate the relationships between serum HE4 (Human Epididymis Protein 4) and CA 125 levels with lymph node metastasis in endometrial cancer. Additionally, it sought to assess the diagnostic value of these biomarkers when combined with enhanced CT imaging to predict lymph node involvement in patients with endometrial cancer.

## Methods and Materials:

## Research subjects:

A retrospective analysis was performed on patients who were diagnosed with endometrial cancer and were hospitalized for surgery in the Department of Obstetrics & Gynaecology at Maa Vindhyavasini Autonomous State Medical College, Mirzapur, India from July 2023 to June 2024. A total of 326 patients constituted the experimental group and were subsequently categorized based on postoperative pathological findings into a lymph node-positive group (53 patients, comprising 22 cases in the premenopausal group and 31 cases in the postmenopausal group) and a lymph node-negative group (273 cases, consisting of 74 cases in the premenopausal group and 199 cases in the postmenopausal group). Additionally, 98 individuals with normal physical examination results from the physical examination center of our hospital were selected as the control group, with 40 individuals in the premenopausal group and 58 in the postmenopausal group.

In the premenopausal group, the average age of patients in the experimental group was 46.73 ± 6.02 years, while in the control group, it was 45.47 ± 7.35 years. In the postmenopausal group, the average age of patients in the experimental group was 59.37 ± 6.39 years and in the control group, it was 59.31 ± 7.82 years, suggesting no significant statistical differences in age between the groups. Our specific inclusion criteria were as follows: (1) pathological confirmation of endometrial cancer; (2) CT scan was performed before the operations; (3) preoperative detection of serum CA125 and HE4; (4) planned extra fascial hysterectomy, sub-extensive hysterectomy or extensive hysterectomy, double appendectomy, or pelvic with/without para-aortic lymph node dissection, to obtain complete postoperative pathological data. The exclusion criteria were as follows: (1) post-hysterectomy and treatment in our hospital; (2) surgical contraindications; (3) preoperative neoadjuvant chemotherapy or radiotherapy; (4) additional previous or concurrent malignant tumor diseases, such as lung cancer, breast cancer, or pancreatic cancer; (5) liver or kidney abnormalities that might affect the levels of HE4; and (6) non-ovarian diseases that increase serum CA125, such as immune system diseases, pleurisy and pericarditis. All subjects were informed about the study before joining and participated in the research voluntarily. The research was approved by the ethics committee.

## Detection methods:

Before treatment, 3 mL of venous blood was collected from all enrolled patients. After the separation of serum, the levels of serum HE4 and CA125 were determined with an i2000 chemiluminescent particle immunoassay (Abbott Diagnostics, Chicago, America). The normal reference value ranges of the kits were CA125 <35 U/ml; HE4, premenopausal <70 pmol/L, postmenopausal <140 pmol/L.

## Enhanced CT examination:

All patients underwent full-abdomen enhanced CT examination before treatment. The CT inspection equipment was a Brilliancei 256-row spiral CT produced by Philips of the Netherlands, and the contrast enhancement agent was iohexol (100 ml). For lymph nodes, CT was used to diagnose lymph node metastasis by scanning the largest axis >10 mm or with central necrosis.

## Statistical processing methods:

The statistical software SPSS (Version 22.0; SPSS Inc, Chicago, Ill) was utilized for analysis. For measurement data not following a normal distribution, median and interquartile range were used for description. The Mann-Whitney U test compared differences between non-normally distributed datasets, while the Kruskal-Wallis H test assessed differences among multiple groups.

Counted data were compared using Pearson's X2 test or Fisher's exact test. Factors exhibiting significant differences were further examined in multivariate logistic regression analysis. Receiver operating characteristic (ROC) curve analysis evaluated the predictive value of HE4 and CA125 for lymph node metastasis. A significance level of p < 0.05 was applied to all statistical analyses.

## Results:

## Serum HE4 and CA125 in relation to lymph node metastasis in endometrial cancer:

Compared with those in the control group, the medians of CA125 and HE4 in each experimental group were significantly higher, with P <0.001. The medians of CA125 and HE4 in the lymph node positive group were significantly different from those in the corresponding lymph node negative group, with P <0.001. The positive rate was calculated according to the reference values of the kits for serum HE4 and CA125. HE4 showed no difference in discriminating lymph node metastasis in the premenopausal group; otherwise, there were statistical differences between the other groups. However, except in the premenopausal lymph node positive group, the medians of HE4 (91.8 pmol/l) and CA125 (96.4 U/ml) were higher than the corresponding standard values (70 pmol/l, 35 U/ml); the values in the remaining groups were below or like standard values (Table 1).

## ROC curves of serum HE4 and CA125 in diagnosis of lymph node metastasis:

In Table 1, the median values of CA125 and HE4 in most experimental groups were observed to be lower than the corresponding reference values provided by the kits. ROC curves were generated for serum HE4 and serum CA125. The cut-off values for HE4 and CA125 were determined to be 52.95pmol/L and 69.45 U/ml, respectively, in the premenopausal group, and 69.15pmol/L and 21.45 U/ml, respectively, in the postmenopausal group. The area under the curve (AUC) for HE4 was 0.73 (p = 0.001) and 0.578 (p = 0.164), while the AUC for CA125 was 0.81 (p < 0.001) and 0.671 (p = 0.002) in the premenopausal and postmenopausal groups, respectively (refer to Figure 2 (see PDF) and Figure 1 (see PDF)).

## Serum HE4, CA125, and CT assessment of pathological lymph node metastasis:

The determined cut-off values for serum HE4 and CA125 were utilized to establish evaluation criteria for lymph node metastasis. In the premenopausal group, the serum concentrations were set at 52.95pmol/L for HE4 and 69.45 U/mL for CA125. In the postmenopausal group, these values were 69.15pmol/L for HE4 and 21.45 U/ml for CA125. The calculated positive rates of lymph node metastasis according to different criteria, were 86.4% (HE4), 68.2% (CT), and CA125 (59.1%) in the premenopausal group, and were CA125 (74.2%), HE4 (64.5%) and 35.5% (CT) in the postmenopausal group. The single factors affecting lymph node metastasis were introduced into multivariable logistic regression analysis. Both CA125 and CT were found to be independent diagnostic factors for lymph node metastasis in the two groups. Moreover, the CT evaluation of lymph node metastasis had the largest OR values (48.94 in the premenopausal group and 6.31 in the postmenopausal group). After multivariate logistic regression analysis, the diagnostic value of HE4 decreased, with P values of 0.054 in the premenopausal group and 0.130 in the menopausal group (Table 2).

## Serum HE4 and CA125 complemented enhanced CT in evaluation of the diagnostic efficacy for lymph node metastasis in endometrial cancer:

According to the cut-off value, the serum concentration of HE4 in the premenopausal group was greater than 52.95pmol/l and that of CA125 was greater than 69.45 U/ml. In the postmenopausal group, HE4 was greater than 69.15pmol/l, and CA125 was greater than 21.45 U/ml. These cut-off values were used as the diagnostic criteria to assess lymph node metastasis. To improve the diagnostic efficacy for lymph node metastasis, we designed diagnostic methods combining the results from enhanced CT and serum HE4 and CA125. In a parallel test, if any of the three methods yielded positive results, the patient was diagnosed positive for lymph node metastasis. In a tandem test, if all three methods yielded positive results, the patient was diagnosed positive for lymph node metastasis. In the premenopausal group, the specificity of enhanced CT alone was 93%, and the sensitivity of HE4 and CA125 was 86% and 59%, respectively. The sensitivity was 100% and the negative predictive value was 100% in the parallel test. In the tandem test, the specificity was 100%, and the positive predictive value was 100% (Table 3). In the postmenopausal group, the specificity of enhanced CT alone was 94%, and the sensitivity of HE4 and CA125 was 65% and 74%, respectively. Thus, the sensitivity was 97% and the negative predictive value was 99% in the parallel test. In the tandem test, the specificity was 97%, and the positive predictive value was 45% (Table 3).

## Discussions:

The study findings suggest that serum CA125 and HE4 are valuable for diagnosing endometrial cancer. Median values in the experimental group were significantly higher than those in the control group, and in the lymph node metastasis group compared to the non-metastasis group. While serum HE4 showed no significant difference in diagnosing lymph node metastasis in the postmenopausal group, both CA125 and HE4 were valuable in diagnosing endometrial cancer and evaluating lymph node metastasis in other groups. Moreover, ROC curve analysis revealed cut-off values for serum HE4 and CA125, which were 52.95pmol/L and 69.45 U/ml in the premenopausal group and 69.15pmol/L and 21.45 U/ml in the postmenopausal group, respectively. Evaluating lymph node metastasis using these cut-off values showed that the diagnostic coincidence rate of CA125 and HE4 was either higher or comparable to that of enhanced CT. Logistic regression analysis indicated that CT had the highest diagnostic value, followed by serum CA125. Combining all three indicators optimized the diagnostic efficiency for lymph node metastasis. Parallel diagnosis improved sensitivity and negative predictive value, while tandem diagnosis improved specificity and positive predictive value. The preoperative evaluation of lymph node metastasis of endometrial cancer has great clinical value for guiding surgery. At present, CT diagnosis remains the most used method, but the greatest limitation of imaging diagnosis is mainly its subjective diagnosis. Razumilava and Blechacz have found that the sensitivity of CT in the diagnosis of lymph node metastasis of endometrial cancer is only 30%-50% [12-13]. Our data also suggested that the positive coincidence rate of CT diagnosis was only 68.2%, but was much lower in the postmenopausal group, at 35.5%. Optimization of diagnostic methods to improve the diagnostic efficiency of lymph node metastasis of endometrial cancer is greatly needed. The development of tumor biomarkers has long been a hotspot in the field of cancer research. Notably, biomarkers have played an important role in the diagnosis, evaluation and follow-up of malignant tumors, because of their low cost and high reproducibility. However, no specific marker has been identified for endometrial cancer to date. In 1984, Niloff *et al.* first proposed that increased serum CA125 is associated with disease progression and can be used in the evaluation of endometrial cancer [14]. However, the diagnostic value of serum CA125 in endometrial cancer has not been widely recognized and applied. Obtaining a pathological diagnosis of endometrial cancer before surgery is relatively easy. Therefore, the diagnostic value of serum CA125 in endometrial cancer has not been fully recognized. Kotowicz *et al.* have reported that CA125 is highly expressed in endometrial cancer, and therefore, not only has diagnostic value, but also aids in disease evaluation, because its expression increases in lymph node metastasis [15].

We reached similar conclusions in this retrospective analysis. Moreover, after obtaining the cut-off value from the ROC curve of CA125, we found that the coincidence rate of the diagnosis of lymph node metastasis was greater than or close to that of CT diagnosis. Therefore, serum CA125 is more valuable as a biomarker to evaluate endometrial cancer, especially lymph node metastasis, than as a diagnostic tool. HE4 is a member of the WFDC domain family, which has received attention in recent years. As a diagnostic marker for ovarian cancer, it has been widely used in clinics. Moreover, HE4 is positively correlated with CA125, and thus the diagnostic value of the two biomarkers in combination in endometrial cancer has also attracted attention in recent years. Both Wang and Antonsen have reported that elevated levels of serum CA125 and HE4 levels can be used in the diagnosis and evaluation of endometrial cancer, especially lymph node metastasis [7, 16]. Our data also suggested that the positive rate and the median value of CA125 and HE4 in endometrial cancer were significantly higher than those in the control group. Moreover, the positive rate and the median value of CA125 and HE4 in the lymph node metastasis group also increased significantly, thus indicating that CA125 and HE4 have value in the diagnosis of endometrial cancer and in the evaluation of lymph node metastasis. Another phenomenon attracted our attention: the median values of CA125 and HE4 in the endometrial cancer group did not increase significantly. The standard values of CA125 and HE4 are 35 U/ml and 70pmol/l, respectively; however, although the median values of CA125 and HE4 in the premenopausal group (96.4 U/ml and 91.8pmol/l, respectively) were higher than the standard value of the kit, the medians in the other groups were lower than the standard values. After further analyzing the data by using either the standard values of the kit or the cut-off values to determine the positive coincidence rate of lymph node metastasis, we found that the positive coincidence rate of lymph node metastasis, evaluated by the cut-off value, was higher than or close to the standard value. These results suggested that because of the existing diagnostic standard values, clinicians may have underestimated the diagnostic value of CA125 and HE4 in lymph node metastasis of endometrial cancer. In this study, the determined cut-off value for serum CA125 for diagnosing lymph node metastasis was 69.45 U/ml in the premenopausal group and 21.45 U/ml in the postmenopausal group. Additionally, the cut-off value for serum HE4 was found to be 52.95pmol/L in the premenopausal group and 69.15pmol/L in the postmenopausal group.

All these values are lower than the standard values provided by the kits, a result similar to those in other reports. Yildiz *et al.* have reported a cut-off value for diagnosis of lymph node metastasis using serum CA125 of 20 U/mL [17]. Dobrzycka has reported an HE4 cut-off value of 78pmol/L for evaluating lymph node metastasis of endometrial cancer [18]. Therefore, the standard values of serum CA125 and HE4 in the evaluation of endometrial cancer are worth discussing. Analysis of lymph node metastasis by using cut-off values shows that, although comprehensive evaluation of CT is the most valuable, use of serum CA125 and HE4 together with CT significantly improves the evaluation efficiency. In the parallel test using the three methods, both the sensitivity and the negative predictive value were 100% in the premenopausal group, whereas the sensitivity was 97% and the negative predictive value was 99% in the postmenopausal group. In the tandem test using the three methods, both the specificity and the positive predictive value reached up to 100% in the premenopausal group and specificity was 96% in the postmenopausal group. Both the parallel test and the tandem test using the three methods improved the diagnostic efficiency. The positive predictive value of the postmenopausal group in the tandem test did not substantially improve, possibly because of the low coincidence rate of CT diagnosis of postmenopausal lymph node metastasis, thus again indicating the subjective limitations of CT diagnosis. The integration of serum CA125, HE4, and MRI can significantly enhance the accuracy of early diagnosis of malignant ovarian tumors, enabling better medical management and treatment for patients [19]. Serum HE4 and CA125 have shown improved effectiveness over existing methods for risk stratification of endometrioid carcinomas, highlighting the need for further investigation [20]. Serum HE4, more than CA125, shows promise as a diagnostic biomarker for endometrial cancer and is associated with markers of disease severity that could assist in pre-operative staging. However, larger prospective studies are needed to validate these findings and establish cancer-specific thresholds [21].

## Conclusion:

Serum CA125 and HE4, when combined with enhanced CT, play a significant role in assessing lymph node metastasis in endometrial cancer. The cut-off reference values for serum CA125 in premenopausal and postmenopausal groups were 69.45 U/ml and 21.45 U/ml, respectively. Similarly, for serum HE4, the cut-off references in the premenopausal and postmenopausal groups were 52.95pmol/L and 69.15pmol/L, respectively. These reference values provide crucial guidance for clinicians in interpreting test results and making informed decisions regarding the management of endometrial cancer patients.

## Author Contributions:

Writing Original Draft, Vandana Maurya, Shruti Singh, Shubham Singh, Shashi Prabha Singh Investigation, Vandana Maurya, Shruti Singh, Shubham Singh, Shashi Prabha Singh Software, Manish Kumar Verma, Shashi Prabha Singh Supervision, Vandana Maurya, Shruti Singh, Shubham Singh, Shashi Prabha Singh, writing-review and editing, Shashi Prabha Singh, Manish Kumar Verma, All authors have read and agreed to the published version of the manuscript.

## Consent for publication:

All authors have declared that no financial support was received from any organization for the submitted work.

## Funding:

No funding was received for this research.

## Figures and Tables

**Table 1:** Serum HE4, CA125 levels and CT in cases and controls

	N	**HE4(n, %, pmol/l)**		**CA125(n, %, U/ml )**	
		**The positive rate M (QL-QU)**		**The positive rate M (QL-QU)**	
Premenopausal	136	37	44.70 (35.15-74.75)	37	23.50 (13.58-36.43)
Lymphnode positive	22	14, 63.6	91.8 (54.5-183.7)	15, 68.2	96.4 (31.5-231.2)
Lymphnode negative	74	23, 31.1	50.9 (39.3-78.7)	22, 29.7	26.7 (17.0-36.9)
Controls	40	0, 0.0	37.4 (31.6-41.8)	0, 0.0	14.0 (10.5-21.3)
P		<0.001	<0.001	<0.001	<0.001
Postmenopausal	288	33	55.10 (39.93-89.18)	64	17.30 (11.40-29.70)
Lymphnode positive	31	6, 19.4*	72.8 (47.2-109.1)	17, 54.8	38.7 (18.5-69.9)
Lymphnode negative	199	27, 13.6*	59.4 (42.2-95.1)	44, 22.1	17.9 (12.0-32.1)
Controls	58	0, 0.0	39.8 (37.6-47.4)	3, 4.7	11.6 (8.9-17.0)
P		0.006	<0.001	<0.001	<0.001

**Table 2:** Logistic regression analysis of postoperative pathological lymph node metastasis and diagnosis of lymph nodes by HE4, CA125 and CT

**Premenopausal**	**Lymph node metastasis**	**Univariate**	**Multivariate**
**HE4**	**Negative (%)**	**Positive (%)**	**OR(95%CI)**	**P**	**OR (95%CI)**	**P**
≥52.95 pmol/L	34 (45.9)	19 (86.4)	7.45 (2.03-27.36)	0.002	7.88 (0.96-64.50)	0.054
<52.95 pmol/L	40 (54.1)	3 (13.6)				
**CA125**						
≥69.45U/ml	4 (5.4)	13 (59.1)	25.28 (6.77-94.45)	<0.001	32.47 (5.10-206.96)	<0.001
<69.45U/ml	70 (94.6)	9 (40.9)				
CT						
+	5 (6.8)	15 (68.2)	29.57 (8.25-105.96)	<0.001	48.94 (7.78-308.03)	<0.001
-	69 (93.2)	7 (31.8)				
**Postmenopausal**						
**HE4**						
≥69.15 pmol/L	82 (41.2)	20 (64.5)	2.59 (1.18-5.71)	0.018	1.94 (0.82-4.58)	0.13
<69.15 pmol/L	117 (58.8)	11 (35.5)				
**CA125**						
≥21.45 U/ml	76 (38.2)	23 (74.2)	4.65 (1.98-10.93)	<0.001	3.15 (1.27-7.81)	0.013
<21.45 U/ml	123 (61.8)	8 (25.8)				
**CT**						
+	12 (6.0)	11 (35.5)	8.57 (3.35-21.92)	<0.001	6.31 (2.34-16.97)	<0.001
-	187 (94.0)	20 (64.5)				

**Table 3:** The diagnostic efficacy of endometrial cancer lymph node metastasis using single or combined cut-off value of HE4, CA125 and enhanced CT

	**SE(%)**	**SP (%)**	PV+(%)	**PV-(%)**
**Premenopausal**				
HE4≥52.95	86	54	36	93
CA125≥69.45	59	95	76	89
Enhanced CT	68	93	75	91
HE4+CA125+Enhanced CT (Parallel test)	100	50	37	100
HE4+CA125+Enhanced CT (Serial test)	27	100	100	82
**Postmenopausal**				
HE4≥69.15	65	17	20	61
CA125≥21.45	74	62	23	94
Enhanced CT	35	94	48	90
HE4+CA125+Enhanced CT(Parallel test)	97	41	20	99
HE4+CA125+Enhanced CT (Serial test)	16	97	45	88
